# Global health inequity and primary care

**DOI:** 10.3399/BJGPO.2024.0189

**Published:** 2024-12-11

**Authors:** Luke N Allen, Luisa M Pettigrew, Josephine Exley, Harry Collin, Shona Bates, Michael Kidd

**Affiliations:** 1 Nuffield Department of Primary Care Health Sciences, University of Oxford, Oxford, UK; 2 Centre for Future Health Systems, University of New South Wales, Sydney, Australia; 3 Department of Health Services Research and Policy, London School of Hygiene & Tropical Medicine, London, UK; 4 Severn School of Primary Care, Bath, UK

**Keywords:** Global Health, Inequity, Primary healthcare

## Introduction

Sadly, inequalities in health are ubiquitous.^1^ Signatories to the 1948 World Health Organization (WHO) constitution committed to promoting *‘health for all’* and this foundational global health principle resonates through the Alma-Ata and Astana Declarations.^2–4^ Universal Health Coverage (UHC) — the contemporary manifestation of *health for all* — is inextricably linked to primary care, as the delivery of at least ten of the 14 ‘tracer’ services being used to monitor UHC progress are critically dependent on well-functioning primary care services.^5^ Primary care services also lie at the centre of the broader philosophical approach of Primary Health Care (PHC), which is underpinned by effective multi-sectoral policy, community engagement, and well-integrated, high quality public health and primary care services.^3,4^ As such, efforts to achieve UHC, deliver PHC, and address health inequities worldwide are fundamentally grounded in the quality and reach of primary care services.^6^

The term ‘primary care’ is often applied broadly and at cross-purposes. It is commonly defined as a model of care that supports five core functions; first-contact, accessible, continuous, comprehensive, and coordinated person-focused health care (Table 1).^7–10^

**Table 1.** Core functions of high quality primary care^8,10^

**First-contact:** the first point of contact for the majority of non-emergency health issues.
**Comprehensiveness:** able to meet the vast majority of health needs in the local community; covering health prevention, health promotion, acute and long-term conditions, rehabilitation, and palliative care.
**Continuity:** provides long-term personal relationships between people and the members of their primary care teams across multiple encounters.
**Coordination:** organises and coordinate service delivery across the entire health system and hold a holistic overview of each service user’s care needs.
**People-centred:** takes a biopsychosocial approach and partners with people as co-decision makers in their care.
**Community-based**: based close to where people live and work and responds to local social determinants of health.

While recent years have witnessed a renewed global focus on primary care, variability in access and quality continues to be a major driver of inequity in health and access to health care worldwide.^5,11,12^ Many of these inequities arise from the social conditions in which people are born, grow, live, work and age and how these affect their ability to access health care.^1^ Work by WHO, the Alliance for Health Policy and Systems Research, the Primary Health Care Performance Initiative, and the Exemplars in Global Health collaborators has produced guidance and frameworks, as well as highlighting good practice to support improved access to high quality primary care.^10,13–17^ However, much remains unknown about the factors underlying successful reforms aimed at addressing health inequities: for instance, which aspects of primary care reform deliver the most cost-effective improvements in health and equity, in what settings, and under what circumstances? These implementation science and health systems research questions are not receiving the attention they deserve in many countries given that extending access to high quality primary care is the cornerstone of UHC.^18^ To begin to address these research questions it is important to examine the different dimensions under which inequities in access to high quality primary care exist.

### Dimensions of disparities in access to high quality primary care

Inequitable access to high quality primary care can be considered across three dimensions: (1) disparities between countries; (2) disparities within countries; and (3) disparities across socioeconomic gradients. While presented separately here, these dimensions may intersect with one another, particularly within countries with fragmented health systems.

#### Disparities between countries

While virtually every country is currently experiencing a shortfall in primary care staff, workforce gaps are not equally shared, with those living in low- and middle-income countries being the least likely to have access to primary care teams and clinicians trained in family medicine.^19,20^ International workforce disparities are compounded by ‘brain drain’, with higher income countries recruiting staff from lower income countries.^21,22^ In settings where there is limited access to primary care, no patient registration, and no routine use of electronic medical records, access to continuity of care is often a luxury.^23–26^ Even in settings where primary care provides the first point of contact, primary care providers may not have the ability to coordinate care across the entire health system, hold responsibility for a person’s care beyond each discrete interaction, or maintain a complete record of each person’s medications and clinical encounters.

There are a number of examples of low- and middle-income countries that have developed high quality primary care systems and introduced effective reforms for at least parts of their populations, including Brazil, Costa Rica, Ethiopia, Ghana, Peru, Sri Lanka, Rwanda, and Thailand.^13,14^ However, many of the world’s best performing care systems are found in higher income country settings, which have the lowest burdens of disease, thus compounding international health inequities.^27^

#### Disparities within countries

Inequity in access to high quality primary care is also manifest within countries through the unequal geographic distribution of primary care facilities and, specifically, the lack of access to qualified primary care providers in many rural areas. Healthcare services tend to be disproportionately located around affluent and well-developed communities while rural, low-income, and socially marginalised communities are often underserved.^28–31^ The reasons for these disparities are complex, but human resources, market forces, and workforce incentives are often central.^1,32^ Encouraging health and care workers to live and practice in the poorest and most remote areas can also be a major challenge.^33^

#### Disparities across socioeconomic gradients

The third dimension is socioeconomic. Irrespective of clinic location or quality, the most disadvantaged individuals in the catchment area tend to face the highest barriers to accessing high quality care.^1^ Again, the issues governing access to high quality care are complex and include a wide range of supply- and demand-side factors, such as approachability, acceptability, affordability, health literacy and values, transport and mobility issues, and structural discrimination.^34^ While access to healthcare services should be driven by need, the reality is that provision of care is often driven by ability to pay in many countries.^35^

### What can be done?

Unless action is taken to improve national primary care systems, the distribution of high quality services within countries, and equitable access to care within each community, there is no chance of ensuring health for all by 2030. Each of these three dimensions is multifaceted and each country’s route to equitable, accessible, and high-quality primary care will be unique.^36^ Here we briefly highlight five important and interlinked supporting elements from the WHO Primary Health Care Measurement Framework: governance and regulation; training and continuous professional development; systems to promote continuity of care; gatekeeping and referral pathways; and funding and payment mechanisms.

#### Governance and regulation

Most health systems have evolved into agglomerations of public, private, and non-profit providers, often delivering overlapping services at variable cost and quality. Governments have a key role to play in regulating the quality of primary care delivered by all provider types.^37^ This requires the establishment of standards and regulatory bodies that can oversee performance with independence and competence. Governance arrangements are also needed to identify priorities for reform — in collaboration with key stakeholders, including primary care providers and users of health services — and subsequently promoted and monitored.^38^ This can be used to stimulate multi-sectoral engagement to plan and deliver services to address the wider determinants of health in partnership with primary care.

### Training and continuous professional development

Specialist training in primary care for community health workers, nurses, doctors, and allied primary care professionals is foundational for the provision of high quality primary care. This includes the training of new professionals and the continuous professional development of existing professionals.^19^ Currently, most of the world’s primary care doctors lack regulated specialist postgraduate training in family medicine and requirements for structured continuous professional development.^39^ Their training should equip them to work within multidisciplinary teams with clear accountability to collectively manage most of their local community’s health needs, from health promotion to end-of-life care.^20^

### Systems to promote continuity of care

Empanelment and health records are mechanisms that can enable relational, informational and managerial continuity of care, as well as supporting practice management, resource allocation, and health system planning.^24,26,40,41^ Empanelment is *'a continuous, iterative set of processes that identify and assign populations to facilities, care teams, or providers who have a responsibility to know their assigned population and to proactively deliver coordinated primary health care towards achieving universal health coverage'.* ^42^ Empanelment involves identifying the target population, creating a population list with identifiable characteristics (such as name, date of birth, and gender), assigning the individuals to a provider, ensuring both parties are aware of their mutual association, and reviewing the assigned lists regularly. Empanelment enables primary care teams to plan and deliver appropriate care for their population, while facilitating continuity of care, ownership over coordinating care, and a shift from reactive to proactive health care.^43^ Multiple mechanisms to improve empanelment are needed to produce a lasting impact, such as financial incentives for both primary care providers and patients and policies that allow primary care services to be adapted to local needs,^26^ while highlighting to both parties how it contributes to continuity of care. Likewise, investment and incentives may be required to support the introduction of health records to facilitate information collection and sharing with the patient, within the primary care team, and with other health providers.

### Gatekeeping and referral pathways

Systems that enable and standardise communication between primary care and other areas of the health system, referrals pathways, and counter-referrals (that is, referrals and communications back to primary care) are required as the building blocks of care coordination.^44^ First-contact or ‘gatekeeper’ reforms to channel access to secondary care through primary care are important, but will not be accepted by the public without trust, which must be earned by consistently demonstrating the delivery of competent, safe, and high quality care.^44^ Gatekeeping is associated with lower patient satisfaction, as it restricts choice, but with better quality of care, lower healthcare use, and lower healthcare expenditure.^45^ Clear referral pathways help to standardise care for patients with similar conditions, and counter-referral letters help to promote management continuity and primary care coordination.^23^

### Funding and payment mechanisms

Currently, around 5–10% of national health spending goes towards primary care in many countries; vastly less than the proportion of total health care that primary care is expected to deliver.^46,47^ Expenditure on primary care must be monitored to ensure investments in high value and cost-effective aspects of primary care are prioritised, many of which can reduce secondary care costs.^48^ Adequate facilities, supplies, and support staff are essential for multidisciplinary primary care teams to operate effectively and efficiently. Blended payment mechanisms, including consideration of inclusion of capitation and pay-for-performance measures, can support greater delivery of health promotion, disease prevention, chronic disease management and community-focused care.^46,49^ Such payment mechanisms may require other systems, such as empanelment, to be in place to both model costings and organise payments.^40,43,50^

Finally, countries seeking to address health inequalities should identify and respond to the needs of communities that face the highest levels of marginalisation and socioeconomic deprivation.^51^ Financial and other incentives can be used to achieve a balanced distribution of health workers.^33,52^ Public services need to reach everyone irrespective of their ability to pay.^53^ In many countries, to ensure equitable access to care, insurance schemes and national health financing systems may need to meet all costs associated with accessing primary care.

### Conclusion: The neglected bedrock of global health equity

In this analysis we have outlined how inequities in health and access to healthcare play out internationally, nationally, and across socioeconomic groups. We present a selection of reforms that can potentially promote the availability of high quality primary care. There is an urgent need for well-funded, scientifically rigorous research into the planning, implementation, and evaluation of primary care reforms in each country, as every context is different.

Most health care is accessed — well or poorly — in community settings by services described as primary care. The differential quality, distribution, and accessibility of these services is an important driver of national and international health inequities. Ensuring primary care can provide first-contact, comprehensive, continuous, coordinated, people-centred, and community-based care is imperative. National governance, workforce, financing, and planning reforms are central to strengthening and rationalising the weak, fragmented, and often ineffective primary care services that currently serve much of the world’s population. All countries — even high-income countries with mature primary care systems —have work to do.

## Data

N/A
